# Avian influenza overview June–August 2026

**DOI:** 10.2903/j.efsa.2026.10369

**Published:** 2026-09-30

**Authors:** European Food Safety Authority (EFSA), European Centre for Disease Prevention and Control (ECDC), European Union Reference Laboratory for Avian Influenza (EURL), Cyril Barbezange, Mariette Ducatez, Alice Fusaro, Jose L. Gonzales, Thijs Kuiken, Raffaella Melilli, Gražina Mirinavičiūtė, Karl Ståhl, Christoph Staubach, Olov Svartström, Calogero Terregino, Katriina Willgert, Evelyn Alarcón, Lisa Kohnle

**Keywords:** avian influenza, captive birds, HPAI, humans, monitoring, poultry, wild birds

## Abstract

Between 5 June and 28 August 2026, 110 highly pathogenic avian influenza (HPAI) A(H5N1) virus detections were reported in domestic (7) and wild (103) birds in 12 countries in Europe. The number of detections remained at a seasonal low throughout the summer, continuing the decline observed since spring. In contrast to previous years, fewer colony‐breeding seabirds were affected, and the geographical range of detections was more limited. Further sporadic detections of HPAI A(H5) virus were reported in wild terrestrial carnivores and pinnipeds. Outside Europe, the epidemic continued in the Americas, where also the HPAI A(H7N3) subtype was detected in Mexico. Following its first introduction to mainland Australia, HPAI A(H5N1) virus spread within local wild bird populations and spilled over to terrestrial carnivores and marine mammals. In the US, the number of dairy cattle farms reportedly affected by HPAI A(H5N1) increased, while detections in captive American minks were reported for the first time. Between 5 June and 31 August 2026, 15 cases of avian influenza virus infection were publicly reported in humans (no fatal cases) in three countries and territories: Bangladesh (one A(H5N1) case), Cambodia (one A(H5N1) case) and China (13 A(H9N2) cases). All human cases reported exposure to poultry or a poultry environment prior to detection or onset of illness. Human infections with avian influenza viruses remain rare and no sustained human‐to‐human transmission has been documented. The risk posed by avian influenza A(H5N1) clade 2.3.4.4b viruses currently circulating in Europe remains low for the general public in the European Union/European Economic Area (EU/EEA) and low‐to‐moderate for those occupationally or otherwise exposed to infected animals or contaminated environments.

## Graphical abstract

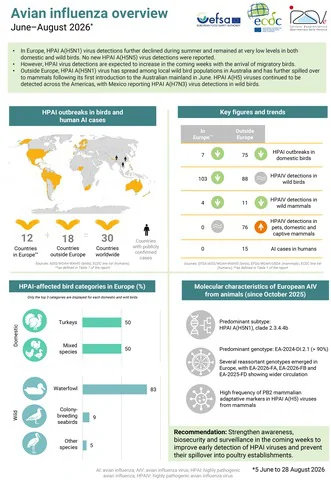

## References

[efs210369-bib-0001] ABC, online‐a . Suspected bird flu infection in fox sparks fears of 'long‐distance' spread. Available online: https://www.abc.net.au/news/2026-08-26/red-fox-suspected-of-h5n1-infection-in-adelaide-suburb/107079798 [Accessed: 20 September 2026]

[efs210369-bib-0002] ABC, online‐b . First H5N1 bird flu detection confirmed in Australian dolphin. Available online: https://www.abc.net.au/news/2026-08-28/sa-detects-h5n1-bird-flu-in-goolwa-dolphin/107090806 [Accessed: 20 September 2026]

[efs210369-bib-0003] Abolnik, C. (2025). Avian influenza situation report—Africa. Canadian Journal of Microbiology, 71, 1–4. 10.1139/cjm-2025-0199 41283637

[efs210369-bib-0004] Agriculture Victoria, online . H5 bird flu update: new detections in wild birds. Available online: https://agriculture.vic.gov.au/about/media-centre/media-releases/2026-releases/h5-bird-flu-update-new-detections-in-wild-birds [Accessed: 20 September 2026]

[efs210369-bib-0005] An, B. M. , Nam, J. H. , Choi, Y. J. , & Kim, E. J. (2026). Serologic Investigation of Poultry Farm Workers Associated with Highly Pathogenic Avian Influenza A(H5N1) Outbreaks in the Republic of Korea during the 2024–2025 season. Public Health Weekly Report, 19(25), 1005–1017. 10.56786/phwr.2026.19.25.1 PMC13327822 42404252

[efs210369-bib-0006] Australian Government, online . Bird flu (avian influenza). Available online: https://www.agriculture.gov.au/campaigns/birdflu/latest-data#event_data [Accessed: 20 September 2026]

[efs210369-bib-0007] Avian Flu Diary , online. Two Reports on HPAI H5N8 Infecting Marine Mammals (Denmark & Germany). Available online: https://afludiary.blogspot.com/2022/02/two-reports-on-hpai-h5n8-infecting.html [Accessed: 28 November 2025]

[efs210369-bib-0008] Ärzteblatt , online. Bird flu virus detected in dead seals. Available online: https://www.aerzteblatt.de/nachrichten/127460/Vogelgrippevirus-bei-toten-Seehunden-nachgewiesen [Accessed: 28 November 2025]

[efs210369-bib-0009] Ballmann, M. Z. , Germeraad, E. A. , Mars, M. H. , Santman‐Berends, I. , & de Boer, S. M. (2026). Serologische screening van rundvee op antilichamen tegen aviaire influenza virus. (Wageningen Bioveterinary Research report; No. 2608033). *Wageningen Bioveterinary* Research 10.18174/713793

[efs210369-bib-0010] Banyard, A. C. , Bennison, A. , Byrne, A. M. P. , Reid, S. M. , Lynton‐Jenkins, J. G. , Mollett, B. , De Silva, D. , Peers‐Dent, J. , Finlayson, K. , Hall, R. , Blockley, F. , Blyth, M. , Falchieri, M. , Fowler, Z. , Fitzcharles, E. M. , Brown, I. H. , & James, J. (2024). Detection and spread of high pathogenicity avian influenza virus H5N1 in the Antarctic Region. Nature Communications, 15(1), 7433. 10.1038/s41467-024-51490-8 PMC11372179 39227574

[efs210369-bib-0011] Beacon, online . HPAI detected on a commercial poultry farm in Santa Bárbara Department, Honduras, amid concurrent cases in wild birds. Available online: https://beaconbio.org/en/report/?reportid=e769ed73-0ff5-4f6c-948a-620713154466&eventid=3a6185f2-acf0-4692-b8dc-137bd1b2e3e7 [Accessed: 20 September 2026]

[efs210369-bib-0012] Bennison, A. , Adlard, S. , Banyard, A. C. , Blockley, F. , Blyth, M. , Browne, E. , Day, G. , Dunn, M. J. , Falchieri, M. , Fitzcharles, E. , Forcada, J. , Davidson, J. F. , Fox, A. , Hall, R. , Holmes, E. , Hughes, K. , James, J. , Lynton‐Jenkins, J. , Marshall, S. , … Phillips, R. A. (2024). A case study of highly pathogenic avian influenza (HPAI) H5N1 at Bird Island, South Georgia: the first documented outbreak in the subantarctic region. Bird Study, 1–12. 10.1080/00063657.2024.2396563

[efs210369-bib-0013] Billias, N. , D'Alessandro, V. , Pouliopoulou, D. V. , Wong, J. J. , Miller, E. , Hopkins, J. P. , Boutsikari, E. C. , Cipriano, L. E. , Da Veiga Pereira, T. , Johnstone, J. , Stranges, S. , Weese, J. S. , MacDermid, J. C. , Quinn, K. L. , Fisman, D. N. , & Bobos, P. (2026). Mapping Reported Modes of Transmission of Highly Pathogenic Avian Influenza A (H5N1) to Humans: A Scoping Review. One Health, 23, 101492. 10.1016/j.onehlt.2026.101492 PMC13393788 42494546

[efs210369-bib-0014] CBC , online. Fort Smith herring gull found with bird flu. Available online: https://www.cbc.ca/news/canada/north/bird-flu-n-w-t-9.7264992 [Accessed: 20 September 2026]

[efs210369-bib-0015] CDC (Centers for Disease Control and Prevention) , online. A(H5) Bird Flu: Current Situation. Available online: https://www.cdc.gov/bird-flu/situation-summary/index.html [Accessed: 20 September 2026]

[efs210369-bib-0016] Central News Agency , online. Avian flu outbreak at quail farm in Fuxing Township, Changhua, culling over 100,000 birds. Available online: https://www.cna.com.tw/news/aloc/202606230270.aspx [Accessed: 20 September 2026]

[efs210369-bib-0017] CFIA (Canadian Food Inspection Agency) , online. Highly pathogenic avian influenza dashboards. Available online: https://inspection.canada.ca/en/animal-health/terrestrial-animals/diseases/reportable/avian-influenza/latest-bird-flu-situation/hpai-dashboards [Accessed: 20 September 2026]

[efs210369-bib-0018] Channuoi , online. The Department of Livestock and Veterinary Health requests to strengthen the prevention and control of avian influenza epidemics nationwide. Available online: https://nhachannuoi.vn/cuc-chan-nuoi-va-thu-y-yeu-cau-tang-cuong-cong-tac-phong-chong-dich-benh-cum-gia-cam-tren-toan-quoc/ [Accessed: 20 September 2026]

[efs210369-bib-0019] Chen, S. , Yang, X. , Qiu, J. , Zhao, J. , Liu, R. , Deng, P. , Xie, Q. , & Zhou, Y. (2026). Epidemiological and clinical characterization of human infections with H9N2 avian influenza virus in Changsha city, China, 2015‐2025. Journal Of Public Health Research, 15(3), 22799036261470475. 10.1177/22799036261470475 PMC13459667 42583221

[efs210369-bib-0020] Chilevision , online. Avian flu in Los Ríos: 241 birds have died and Senapred maintains preventive alert. Available online: https://www.chilevision.cl/noticias/nacional/gripe-aviar-en-los-rios-241-aves-han-muerto-y-senapred-mantiene-alerta-preventiva/ [Accessed: 20 September 2026]

[efs210369-bib-0021] CHP (Centre for Health Protection) , online‐a. Avian Influenza Report. Reporting period: June 7, 2026 – June 13, 2026 (Week 24). Available online: https://www.chp.gov.hk/files/pdf/2026_avian_influenza_report_vol22_wk24.pdf [Accessed: 20 September 2026a]

[efs210369-bib-0022] CHP (Centre for Health Protection) , online‐b. Avian Influenza Report. Reporting period: August 23, 2026 – August 29, 2026 (Week 35). Available online: https://www.chp.gov.hk/files/pdf/2026_avian_influenza_report_vol22_wk35.pdf [Accessed: 20 September 2026b]

[efs210369-bib-0023] CNN Brasil , online. New Zealand reports first case of H5N1 avian influenza in a seabird. Available online: https://www.cnnbrasil.com.br/internacional/nova-zelandia-registra-primeiro-caso-de-gripe-aviaria-h5n1-em-ave-marinha/ [Accessed: 20 September 2026]

[efs210369-bib-0024] Communicable Disease Control Department, Ministry of Health Cambodia , online. Press release on a 9‐month‐old child with avian influenza (H5N1). Available online: https://www.cdcmoh.gov.kh/977-h5n1-09072026 [Accessed: 20 September 2026]

[efs210369-bib-0025] Danviet , online. A commune of Ninh Binh province had to destroy more than 1,100 ducks infected with the avian influenza virus. Available online: https://danviet.vn/mot-xa-cua-tinh-ninh-binh-phai-tieu-huy-hon-1100-con-vit-nhiem-vi-rut-cum-gia-cam-d1434895.html [Accessed: 20 September 2026]

[efs210369-bib-0026] Deng, W. , Xiang, S. , Che, R. , Guo, Q. , Li, S. , Cao, J. , Liu, Z. , Yan, Y. , He, Y. , Zeng, C. , Long, H. , Deng, A. , Li, Z. , & Lu, J. (2026). A cross‐sectional study of avian influenza virus in poultry markets of Guangdong China, 2025. Frontiers In Cellular and Infection Microbiology, 16, 1914072. 10.3389/fcimb.2026.1914072 PMC13503173 42643360

[efs210369-bib-0027] Devdiscourse , online. New Zealand Introduces Mandatory Biosecurity Rules to Combat H5 Bird Flu. Available online: https://www.devdiscourse.com/article/law-order/3956255-new-zealand-introduces-mandatory-biosecurity-rules-to-combat-h5-bird-flu [Accessed: 20 September 2026]

[efs210369-bib-0028] Diario de Yucatán , online. Avian influenza H7N3 and H5N1 in Mexico: More than one million birds will be eliminated in 2026. Available online: https://www.yucatan.com.mx/mexico/2026/08/22/gripe-aviar-h7n3-y-h5n1-en-mexico-eliminan-mas-de-un-millon-de-aves-en-2026.html [Accessed: 20 September 2026]

[efs210369-bib-0029] Du, W. , Dai, M. , Li, Z. , Boons, G. J. , Peeters, B. , van Kuppeveld, F. J. M. , de Vries, E. , & de Haan, C. A. M. (2018). Substrate Binding by the Second Sialic Acid‐Binding Site of Influenza A Virus N1 Neuraminidase Contributes to Enzymatic Activity. Journal of Virology, 92(20), e01243‐18. 10.1128/JVI.01243-18 PMC6158415 30089692

[efs210369-bib-0030] DutchNews , online. Eight kittens die of bird flu, pet owners warned to be alert. Available online: https://www.dutchnews.nl/2025/12/eight-kittens-die-of-bird-flu-pet-owners-warned-to-be-alert/ [Accessed: 28 November 2025]

[efs210369-bib-0031] ECDC (European Centre for Disease Prevention and Control) . 2019 Operational tool on rapid risk assessment methodology. Available online: https://www.ecdc.europa.eu/sites/default/files/documents/operational-tool-rapid-risk-assessment-methodolgy-ecdc-2019.pdf [Accessed: 20 September. 2026]

[efs210369-bib-0032] ECDC (European Centre for Disease Prevention and Control) . 2024 Surveillance and targeted testing for the early detection of zoonotic influenza in humans during the winter period in the EU/EEA. Available here: https://www.ecdc.europa.eu/en/publications-data/surveillance-and-targeted-testing-early-detection-zoonotic-influenza-humans [Accessed: 20 September 2026].

[efs210369-bib-0033] ECDC (European Centre for Disease Prevention and Control) , & EFSA (European Food Safety Authority) . (2026). Surveillance of West Nile virus infections in humans and animals in Europe, monthly report – data submitted up to 3 September 2026. Available online: https://www.ecdc.europa.eu/en/infectious-disease-topics/west-nile-virus-infection/surveillance-and-disease-data/monthly-updates [Accessed: 20 September 2026].

[efs210369-bib-0034] EFSA (European Food Safety Authority) , ECDC (European Centre for Disease Prevention and Control) , EURL (European Union Reference Laboratory for Avian Influenza) , Brown, I. , Kuiken, T. , Mulatti, P. , Smietanka, K. , Staubach, C. , Stroud, D. , Therkildsen, O. R. , Willeberg, P. , Baldinelli, F. , Verdonck, F. , & Adlhoch, C. (2017). Avian influenza overview September–November 2017. EFSA Journal, 15(12), e05141. 10.2903/j.efsa.2017.5141 PMC7010192 32625395

[efs210369-bib-0035] EFSA (European Food Safety Authority) , ECDC (European Centre for Disease Prevention and Control) , EURL (European Union Reference Laboratory for Avian Influenza) , Alexakis, L. , Buczkowski, H. , Ducatez, M. , Fusaro, A. , Gonzales, J. L. , Kuiken, T. , Ståhl, K. , Staubach, C. , Svartström, O. , Terregino, C. , Willgert, K. , Delacourt, R. , & Kohnle, L. (2024). Avian influenza overview June–September 2024. EFSA Journal, 22(10), e9057. 10.2903/j.efsa.2024.9057 PMC11492803 39434784

[efs210369-bib-0036] EFSA (European Food Safety Authority) , ECDC (European Centre for Disease Prevention and Control) , EURL (European Union Reference Laboratory for Avian Influenza) , Alexakis, L. , Buczkowski, H. , Ducatez, M. , Fusaro, A. , Gonzales, J. L. , Kuiken, T. , Mirinavičiūtė, G. , Ståhl, K. , Staubach, C. , Svartström, O. , Terregino, C. , Willgert, K. , Melo, M. , & Kohnle, L. (2025a). Avian influenza overview June–September 2025. EFSA Journal, 23(10), e9702. 10.2903/j.efsa.2025.9702 PMC12558595 41158675

[efs210369-bib-0037] EFSA (European Food Safety Authority) , ECDC (European Centre for Disease Prevention and Control) , EURL (European Union Reference Laboratory for Avian Influenza) , Alexakis, L. , Buczkowski, H. , Ducatez, M. , Fusaro, A. , Gonzales, J. L. , Kuiken, T. , Mirinavičiūtė, G. , Ståhl, K. , Staubach, C. , Svartström, O. , Terregino, C. , Willgert, K. , Melo, M. , & Kohnle, L. (2025b). Avian influenza overview March–June 2025. EFSA Journal, 23(7), e9520. 10.2903/j.efsa.2025.9520 PMC12284602 40703963

[efs210369-bib-0038] EFSA (European Food Safety Authority) , ECDC (European Centre for Disease Prevention and Control) , EURL (European Union Reference Laboratory for Avian Influenza) , Buczkowski, H. , Ducatez, M. , Fusaro, A. , Gonzalez, J. L. , Kuiken, T. , Mirinavičiūtė, G. , Ståhl, K. , Staubach, S. , Svartström, O. , Terregino, C. , Willgert, K. , Alarcón, E. , & Kohnle, L. (2026a). Avian influenza overview March–May 2026. EFSA Journal, 24(7), e10196. 10.2903/j.efsa.2026.10196 PMC13343291 42421857

[efs210369-bib-0039] EFSA (European Food Safety Authority) , ECDC (European Centre for Disease Prevention and Control) , EURL (European Union Reference Laboratory for Avian Influenza) , Barbezange, C. , Buczkowski, H. , Ducatez, M. , Fusaro, A. , Gonzalez, J. L. , Kuiken, T. , Mirinavičiūtė, G. , Ståhl, K. , Staubach, S. , Svartström, O. , Terregino, C. , Willgert, K. , Alarcón, E. , & Kohnle, L. (2026b). Avian influenza overview December 2025–February 2026. EFSA Journal, 24(3), e10015. 10.2903/j.efsa.2026.10015 PMC13093848 42016297

[efs210369-bib-0040] EFSA AHAW Panel (EFSA Panel on Animal Health and Animal Welfare) , ECDC (European Centre for Disease Prevention and Control) , Alvarez, J. , Boklund, A. , Dippel, S. , Dórea, F. , Figuerola, J. , Herskin, M. S. , Michel, V. , Chueca, M. Á. M. , Nannoni, E. , Nielsen, S. S. , Nonno, R. , Riber, A. B. , Stegeman, J. A. , Ståhl, K. , Thulke, H. , Tuyttens, F. , Winckler, C. , Brugerolles, C. , & A Melidou , A. (2025). Preparedness, prevention and control related to zoonotic avian influenza. EFSA Journal, 23(1), e9191. 10.2903/j.efsa.2025.9191 PMC11775931 39882189

[efs210369-bib-0041] EGMP (European Goose Management Platform) Data Centre . (2026). Greylag Goose breeding index [Graph]. Aarhus University. Available online: https://projects.au.dk/fileadmin/projects/can.au.dk/Graagaes/EGMP/Greylag/GGBreedingindex_11042023_fig.pdf [Accessed: 10 September 2026].

[efs210369-bib-0042] El Heraldo , online. SENASA confirms H5N1 avian influenza in wild birds in Lempira. Available online: https://www.elheraldo.hn/honduras/honduras-senasa-confirma-influenza-aviar-h5n1-aves-silvestres-lempira-BD30993992 [Accessed: 10 September 2026]

[efs210369-bib-0043] Está Pasando , online. Authorities confirm new case of avian influenza in Iquique. Available online: https://estapasando.cl/nacional/autoridades-confirman-nuevo-caso-de-influenza-aviar-en-iquique/ [Accessed: 10 September 2026]

[efs210369-bib-0044] European Commission , online. PAFF committees. Available online: https://food.ec.europa.eu/horizontal-topics/committees/paff-committees/animal-health-and-welfare_en [Accessed: 10 September 2026]

[efs210369-bib-0045] FAO (Food and Agriculture Organization of the United Nations) , online. Sub‐Saharan Africa HPAI situation update. Available online: https://www.fao.org/animal-health/situation-updates/sub-saharan-africa-hpai/en [Accessed: 10 September 2026]

[efs210369-bib-0046] FAO (Food and Agriculture Organization of the United Nations) , WHO (World Health Organization) and WOAH (World Organisation for Animal Health) , online‐a. Updated joint FAO/WHO/WOAH public health assessment of recent high pathogenicity avian influenza A(H5) virus events in animals and people. Assessment based on data as of 1 March 2026. Available online: https://www.who.int/publications/m/item/updated-joint-fao-who-woah-public-health-assessment-of-recent-high-pathogenicity-avian-influenza-a(h5)-virus-events-in-animals-and-people [Accessed: 10 September 2026]

[efs210369-bib-0047] FAO (Food and Agriculture Organization of the United Nations) , WHO (World Health Organization) and WOAH (World Organisation for Animal Health) , online‐b. Updated joint FAO/WHO/WOAH public health assessment of recent influenza A(H5) virus events in animals and people. Assessment based on data as of 18 November 2024. Available online: https://www.who.int/publications/m/item/updated-joint-fao-who-woah-assessment-of-recent-influenza-a(h5n1)-virus-events-in-animals-and-people_dec2024 [Accessed: 10 September 2026]

[efs210369-bib-0048] Floyd, T. , Banyard, A. C. , Lean, F. Z. , Byrne, A. M. , Fullick, E. , Whittard, E. , Mollett, B. C. , Bexton, S. , Swinson, V. , Macrelli, M. , Lewis, N. S. , Reid, S. M. , Núñez, A. , Duff, J. P. , Hansen, R. , & Brown, I. H. (2021). Encephalitis and Death in Wild Mammals at a Rehabilitation Center after Infection with Highly Pathogenic Avian Influenza A(H5N8) Virus, United Kingdom. Emerging Infectious Diseases, 27(11), 2856–2863. 10.3201/eid2711.211225 PMC8544989 34670647

[efs210369-bib-0049] Fosse, J. H. , Rømo, G. , Bonfante, F. , Myhrvold, I. K. , Soetart, K. S. , Udjus, K. , & Tønnessen, R. (2025). Detection of Antibodies Specific to H5 Avian Influenza Virus in a Sheep in Norway, June 2024, 11 Months After an Outbreak of Highly Pathogenic Avian Influenza in a Nearby Seabird Colony. Influenza and Other Respiratory Viruses, 19(11), e70182. 10.1111/irv.70182 PMC12626899 41254898

[efs210369-bib-0050] Fusaro, A. , Pu, J. , Zhou, Y. , Lu, L. , Tassoni, L. , Lan, Y. , Lam, T. T. , Song, Z. , Bahl, J. , Chen, J. , Gao, G. F. , Monne, I. , & Liu, J. (2024a). Proposal for a Global Classification and Nomenclature System for A/H9 Influenza Viruses. Emerging Infectious Diseases, 30(8), 1–13. 10.3201/eid3008.231176 PMC11286050 39043566

[efs210369-bib-0051] Fusaro, A. , Zecchin, B. , Giussani, E. , Palumbo, E. , Agüero‐García, M. , Bachofen, C. , Bálint, Á. , Banihashem, F. , Banyard, A. C. , Beerens, N. , Bourg, M. , Briand, F. , Bröjer, C. , Brown, I. H. , Brugger, B. , Byrne, A. M. P. , Cana, A. , Christodoulou, V. , Dirbakova, Z. , … Monne, I. (2024b). High pathogenic avian influenza A(H5) viruses of clade 2.3.4.4b in Europe—Why trends of virus evolution are more difficult to predict. Virus Evolution, 10(1), veae027. 10.1093/ve/veae027 PMC11065109 38699215

[efs210369-bib-0052] G1 Sao Paulo , online. São Paulo confirms case of avian flu in duck in Ibirapuera Park. Available online: https://g1.globo.com/sp/sao-paulo/noticia/2026/08/08/sp-confirma-caso-de-gripe-aviaria-em-marreco-no-parque-ibirapuera.ghtml [Accessed: 10 September 2026]

[efs210369-bib-0053] Gao, X. , Yu, H. , Zhang, N. , Liu, L. , Tong, J. , Qi, X. , Huang, H. , Wang, S. , Li, Z. , Du, Y. , & Zhu, L. (2026). Epidemiological and Virological Characteristics of H9N2 Avian Influenza Virus in Jiangsu Province, China, 2024. Viruses, 18(6), 687. 10.3390/v18060687 PMC13307689 42357696

[efs210369-bib-0054] GISAID , online. Available online: https://gisaid.org/ [Accessed: 10 September 2026]

[efs210369-bib-0055] Github izsvenezie‐virology , online‐a. FlutMut. Available online: https://github.com/izsvenezie-virology/FluMut [Accessed: 10 September 2026a]

[efs210369-bib-0056] Github izsvenezie‐virology , online‐b. FlutMutDB with v5.0 mutations. Available online: https://github.com/izsvenezie-virology/FluMutGUI/releases/tag/v.3.0.0 [Accessed: 10 September 2026b]

[efs210369-bib-0057] Giussani, E. , Sartori, A. , Salomoni, A. , Cavicchio, L. , De Battisti, C. , Pastori, A. , Varotto, M. , Zecchin, B. , Hughes, J. , Monne, I. , & Fusaro, A. (2025). FluMut: a tool for mutation surveillance in highly pathogenic H5N1 genomes. Virus Evolution, 11, veaf011. 10.1093/ve/veaf011 PMC11908534 40093844

[efs210369-bib-0058] Golke, A. , Jańczak, D. , Szaluś‐Jordanow, O. , Dzieciątkowski, T. , Sapierzyński, R. , Moroz‐Fik, A. , Mickiewicz, M. , & Frymus, T. (2024). Natural Infection with Highly Pathogenic Avian Influenza A/H5N1 Virus in Pet Ferrets. Viruses, 16, 931. 10.3390/v16060931 PMC11209192 38932223

[efs210369-bib-0059] GovUK (UK Health Security Agency) , online‐a. Influenza of avian origin confirmed in a sheep in Yorkshire. Available online: https://www.gov.uk/government/news/influenza-of-avian-origin-confirmed-in-a-sheep-in-yorkshire [Accessed: 28 November 2025a]

[efs210369-bib-0060] GovUK (UK Health Security Agency) , online‐b. Confirmed findings of influenza of avian origin in captive mammals. Available online: https://www.gov.uk/government/publications/bird-flu-avian-influenza-findings-in-captive-mammals/confirmed-findings-of-influenza-of-avian-origin-in-captive-mammals [Accessed: 28 November 2025b]

[efs210369-bib-0061] GovUK (UK Health Security Agency) , online‐c. Bird flu (avian influenza): latest situation in England. Available online: https://www.gov.uk/guidance/bird-flu-avian-influenza-latest-situation-in-england#risk [Accessed: 10 September 2026]

[efs210369-bib-0062] Günther, A. , Wassermann, J. , Heck, J. , Bussi, M. , Aebischer, A. , Staubach, C. , Bergmann, H. , Leendertz, F. H. , Beer, M. , Schares, G. , & Wernike, K. (2026). Serologic surveillance of highly pathogenic Avian influenza virus subtype H5 in wildlife, Northeast Germany, 2023–2025. Emerging Infectious Diseases, 32(5), 790–793. 10.3201/eid3205.251555 PMC13175092 42044493

[efs210369-bib-0063] Guo, Y. , Shu, S. , Zhou, Y. , Peng, W. , Jiang, Z. , Li, Y. , Li, T. , Du, F. , Wang, L. , Chen, X. , Dong, J. , Zhao, C. , Wang, M. H. , Sun, Y. , Sun, H. , Lu, L. , Digard, P. , Chang, K. , Yen, H. , … Pu, J. (2025). An emerging PB2‐627 polymorphism increases the zoonotic risk of avian influenza virus by overcoming ANP32 host restriction in mammalian and avian hosts. Journal of Virology, 99(9), e00853‐25. 10.1128/jvi.00853-25 PMC12455948 40862614

[efs210369-bib-0064] Halford, F. , Blomquist, P. B. , Bray, N. , Byers, C. , Inzoungou‐Massanga, C. , Rees, C. , Waghorn, M. , Awokoya, M. , Lunt, R. , Cunningham, N. , Chapman, M. , Jones, P. , Machin, N. , Chalikias, G. , Lord, A. , Benee, R. , Makki, S. , Kele, B. , Charlett, A. , … Puleston, R. (2026). A cohort study of persons exposed to highly pathogenic avian influenza A(H5N1) at premises with infected animals, England, 2023 to 2025. Euro Surveillance, 31(31). 10.2807/1560-7917.es.2026.31.31.2500906 42565266

[efs210369-bib-0065] Hamunyela, E. , Coetzee, L. , Marcacci, M. , Ancora, M. , Celani, P. , Secondini, B. , Mincarelli, L. , Monne, I. , Dianati, M. , & Molini, U. (2026). Highly pathogenic avian influenza H5N1 virus outbreak among common terns (Sterna hirundo) in Namibia, 2025‐2026. Veterinaria Italiana, 62(2). 10.12834/VetIt.4031.40792.4 42237945

[efs210369-bib-0066] Hervé, S. , Schmitz, A. , Briand, F. , Gorin, S. , Quéguiner, S. , Niqueux, É. , Paboeuf, F. , Scoizec, A. , Bouquin‐Leneveu, S. L. , Eterradossi, N. , & Simon, G. (2021). Serological Evidence of Backyard Pig Exposure to Highly Pathogenic Avian Influenza H5N8 Virus during 2016‐2017 Epizootic in France. Pathogens, 10. 10.3390/pathogens10050621 PMC8158469 34070190

[efs210369-bib-0067] Hindustan Times , online. All zoos in U.P, Etawah lion safari shut after bird flu scare. Available online: https://www.hindustantimes.com/cities/lucknow-news/all-zoos-in-u-p-etawah-lion-safari-shut-after-bird-flu-scare-101747164596761.html [Accessed: 28 November 2025]

[efs210369-bib-0068] Hondudiario , online. SENASA finds 320 wild birds dead from avian influenza in Lempira and Copán. Available online: https://www.hondudiario.com/2026/06/16/senasa-encuentra-320-aves-silvestres-muertas-por-influenza-aviar-en-lempira-y-copan/ [Accessed: 10 September 2026]

[efs210369-bib-0069] Infobae , online‐a. First outbreak of avian flu confirmed in Reque and 54 backyard birds will be slaughtered as part of SENASA's sanitary protocol. Available online: https://www.infobae.com/peru/2026/07/14/confirman-primer-brote-de-gripe-aviar-en-reque-y-sacrificaran-54-aves-de-traspatio-como-parte-del-protocolo-sanitario-de-senasa/ [Accessed: 10 September 2026a]

[efs210369-bib-0070] Infobae , online‐b. New outbreak of H5N1 avian flu in Peru: why SENASA declared a health emergency and what you should know about this disease. Available online: https://www.infobae.com/peru/2026/07/14/nuevo-brote-de-la-gripe-aviar-h5n1-en-peru-por-que-el-senasa-declaro-emergencia-sanitaria-y-lo-que-debes-saber-de-esta-enfermedad/ [Accessed: 10 September 2026b]

[efs210369-bib-0071] Informa al minuto , online. H5N1 avian influenza maintains two active outbreaks in the Los Ríos Region. Available online: https://informaalminuto.cl/influenza-aviar-h5n1-mantiene-dos-focos-activos-en-la-region-de-los-rios/ [Accessed: 10 September 2026]

[efs210369-bib-0072] Kutkat, O. , Gomaa, M. , Moatasim, Y. , Taweel, A. E. , Kamel, M. N. , Sayes, M. E. , GabAllah, M. , Kandeil, A. , McKenzie, P. P. , Webby, R. J. , Kayali, G. , Ali, M. A. , & El‐Shesheny, R. (2024). Highly pathogenic avian influenza virus H5N1 clade 2.3.4.4b in wild rats in Egypt during 2023. Emerging Microbes & Infections, 13(1), 2396874. 10.1080/22221751.2024.2396874 PMC11382695 39193629

[efs210369-bib-0073] Lee, E. , Lee, Y. , Lee, Y. , Kye, S. , Lewis, N. S. , Brown, I. H. , Sagong, M. , Heo, G. , Kang, Y. , Cho, H. , Kang, H. , Cheon, S. , Lee, M. , Park, B. , Kim, Y. , Lee, Y. , & Lee, Y. (2018). Characterization of a novel reassortant H5N6 highly pathogenic avian influenza virus clade 2.3.4.4 in Korea, 2017. Emerging Microbes & Infections, 7(1), 1–3. 10.1038/s41426-018-0104-3 PMC5997646 29895932

[efs210369-bib-0074] Leguia, M. , Garcia‐Glaessner, A. , Muñoz‐Saavedra, B. , Juarez, D. , Barrera, P. , Calvo‐Mac, C. , Jara, J. , Silva, W. , Ploog, K. , Amaro, L. , Colchao‐Claux, P. , Johnson, C. K. , Uhart, M. M. , Nelson, M. I. , & Lescano, J. (2023). Highly pathogenic avian influenza A (H5N1) in marine mammals and seabirds in Peru. Nature Communications, 14, 5489. 10.1038/s41467-023-41182-0 PMC10484921 37679333

[efs210369-bib-0075] Lin, C. , Tsou, T. , Chu, P. , Yang, S. , Yu, I. , Li, Y. , Liu, M. , & Chih, Y. (2026). Serological Surveillance of Avian Influenza Virus Exposure Using Paired Sera Among Occupational Contacts in Taiwan, January–September 2025. Influenza and Other Respiratory Viruses, 20(7), e70297. 10.1111/irv.70297 PMC13386101 42478106

[efs210369-bib-0076] Madslien, K. , Fosse, J. H. , Aars, J. , Bøe, C. A. , Andersen, M. , Buhler, K. , Fjeldheim, I. , Gjerset, B. , Jørgensen, T. , Myhrvold, I. K. , Rohringer, A. , Sturød, K. , Tryland, M. , Ytrehus, B. , Tønnessen, R. , & Nymo, I. H. (2026). Highly Pathogenic Avian Influenza H5N5 in a Polar Bear and Atlantic Walrus, Svalbard, 2026, with Widespread Seroconversion in Polar Bears. bioRxiv, 2026(7). Advance online publication. 10.64898/2026.07.20.739480

[efs210369-bib-0077] Mongabay , online‐a. Nepal zoo bird flu outbreak raises alarm for national parks. Available online: https://news.mongabay.com/2026/08/nepal-zoo-bird-flu-outbreak-raises-alarm-for-national-parks/ [Accessed: 10 September 2026a]

[efs210369-bib-0078] Mongabay , online‐b. Nepal's Central Zoo faces questions over its bird flu response. Available online: https://news.mongabay.com/2026/06/nepals-central-zoo-faces-questions-over-its-bird-flu-response/ [Accessed: 10 September 2026b]

[efs210369-bib-0079] MPU (Municipalidad Provincial de Utcubamba) , online. Press Release: SENASA REPORTS ON AVIAN INFLUENZA H5N1. Available online: https://www.facebook.com/501027682054882/posts/1482154123942228 [Accessed: 10 September 2026]

[efs210369-bib-0080] News24 , online. Bird flu fears grow after 1000+ mass crested tern deaths in South Australia, 28 dead penguins found on Tasmanian island. Available online: https://www.news24.com.au/latest/28-dead-penguins-found-on-tasmanias-king-island-spark-bird-flu-fears/news-story/fa7b6e8a2e7335d364c24b1c08801061 [Accessed: 10 September 2026]

[efs210369-bib-0081] Norwegian Veterinary Institute , online. Bird flu detected in yet another polar bear in Svalbard. Available online: https://www.vetinst.no/nyheter/fugleinfluensa-pavist-hos-enda-en-isbjorn-pa-svalbard [Accessed: 10 September 2026]

[efs210369-bib-0082] Nyarko, S. O. , Kwasah, L. , Boatemaa, L. , Ntim, N. A. A. , Adusei‐Poku, M. , Sarpong, G. M. , Magnusen, V. , Wutsika, J. , Ago, S. , Amenuvor, E. A. A. , Wordui, J. , Sekyi‐Yorke, A. N. , Takyi, C. , Tackie, R. , Doku, I. K. , Nyarko, J. A. , Quarcoo, J. A. , Kyiire, G. A. , Odoom, T. , & Asante, I. A. (2026). Genetic and Epidemiological Evidence of Avian Influenza A(H9N2) Detection Among Poultry in Ghana, 2022. Viruses, 18(7), 725. 10.3390/v18070725 PMC13431622 42515577

[efs210369-bib-0083] Outbreak News Today , online. Denmark reports 1st highly pathogenic avian influenza case in harbor seal. Available online: http://outbreaknewstoday.com/denmark-reports-1st-highly-pathogenic-avian-influenza-case-in-harbor-seal-89870/ [Accessed: 28 November 2025]

[efs210369-bib-0084] Pang, Z. , Zhong, P. , Mai, C. , Fan, J. , Wu, Y. , Zhang, Y. , Liu, H. , Sun, H. , Sun, H. , Liao, M. , Sun, H. , & Sun, H. (2026). Genetic evolution, phylodynamics, geographic spread of H9N2 avian influenza viruses in China from 2014 to 2025: an increasing potential zoonotic risk. Emerging Microbes & Infections, 15(1), 2713320. 10.1080/22221751.2026.2713320 PMC13470927 42545215

[efs210369-bib-0085] PASTAAF (Préfet Administrateur Supérieur des Terres Australes et Antarctiques Françaises) , online‐a. The mortalities observed in wild animals in Crozet are attributable to highly pathogenic avian influenza (HPAI). Available online: https://taaf.fr/actualite/les-mortalites-constatees-dans-la-faune-sauvage-a-crozet-sont-imputables-a-linfluenza-aviaire-hautement-pathogene-iahp/ [Accessed: 28 November 2025a]

[efs210369-bib-0086] PASTAAF (Préfet Administrateur Supérieur des Terres Australes et Antarctiques Françaises) , online‐b. As in Crozet, the mortalities observed in wildlife in Kerguelen are attributable to highly pathogenic avian influenza (HPAI). Available online: https://taaf.fr/actualite/comme-a-crozet-les-mortalites-constatees-dans-la-faune-sauvage-a-kerguelen-sont-imputables-a-linfluenza-aviaire-hautement-pathogene-iahp/ [Accessed: 28 November 2025b]

[efs210369-bib-0087] Phys.org , online. Antarctic expedition confirms the spread of the highly pathogenic avian influenza virus in the Weddell Sea. Available online: https://phys.org/news/2025-02-antarctic-highly-pathogenic-avian-influenza.html [Accessed: 28 November 2025]

[efs210369-bib-0088] Pinto, R. M. , Bakshi, S. , Lytras, S. , Zakaria, M. K. , Swingler, S. , Worrell, J. C. , Herder, V. , Hargrave, K. E. , Varjak, M. , Cameron‐Ruiz, N. , Rodriguez, M. C. , Varela, M. , Wickenhagen, A. , Loney, C. , Pei, Y. , Hughes, J. , Valette, E. , Turnbull, M. L. , Furnon, W. , … Palmarini, M. (2023). BTN3A3 evasion promotes the zoonotic potential of influenza A viruses. Nature, 619, 338–347. 10.1038/s41586-023-06261-8 37380775

[efs210369-bib-0089] Préfet Des Landes , online. Influenza aviaire hautement pathogène dans la faune sauvage cas détecté sur une loutre dans les Landes. Available online: https://www.landes.gouv.fr/Actualites/Salle-de-presse/Communiques-de-presse/2025/IAHP-dans-la-faune-sauvage-cas-detecte-sur-une-loutre-dans-les-Landes [Accessed: 28 November 2025]

[efs210369-bib-0090] Pulit‐Penaloza, J. , Belser, J. A. , Brock, N. , Kieran, T. J. , Pappas, C. , Zeng, H. , Sun, X. , De la Cruz, J. A. , Hatta, Y. , Di, H. , Davis, C. T. , & Maines, T. R. (2026). Risk assessment of avian influenza A(H5N5) virus from the first human case using the ferret model. Journal of Virology, 100, e0085626. 10.1128/jvi.00856-26 PMC13595979 42578664

[efs210369-bib-0091] Pulso , online. Detectan dos casos de influenza aviar en Zoológico de Chiapas. Available online: https://pulsoslp.com.mx/nacional/casos-de-influenza-aviar-detectados-en-zoomat-chiapas/2062451 [Accessed: 10 September 2026]

[efs210369-bib-0092] Raut, A. A. , Aasdev, A. , Kumar, N. , Pathak, A. , Mishra, A. , Sehgal, P. , Pateriya, A. K. , Pandey, M. , Bhatia, S. , & Mishra, A. (2025). bioRxiv, 2025(2) Advance online publication. 10.1101/2025.02.23.638954

[efs210369-bib-0093] Ren, Y. , Li, H. , Yan, W. , Liu, X. , Chi, W. , Luo, R. , Stoeger, T. , Wajid, A. , Dodovski, A. , Gao, C. , Wang, G. , Lusida, M. I. , Mingala, C. N. , Andreychuk, D. B. , & Yin, R. (2026). Novel Reassortant H9N2 Avian Influenza Viruses with Dual Receptor‐Binding Capacity and Evidence of Direct Mammalian Infectivity Circulating in Northeast China Live Poultry Markets. Viruses, 18(7), 771. 10.3390/v18070771 PMC13431628 42515623

[efs210369-bib-0094] Revilla‐Fernández, S. (2026). AGES (Austrian Agency for Health and Food Safety). Personal communication by e‐mail.

[efs210369-bib-0095] RNZ , online. Bird flu suspected to have spread to seal at key breeding ground off Kangaroo Island. Available online: https://www.rnz.co.nz/news/world/1099389/bird-flu-suspected-to-have-spread-to-seal-at-key-breeding-ground-off-kangaroo-island [Accessed: 10 September 2026]

[efs210369-bib-0096] Rosone, F. , Bonfante, F. , Sala, M. G. , Maniero, S. , Cersini, A. , Ricci, I. , Garofalo, L. , Caciolo, D. , Denisi, A. , Napolitan, A. , Parente, M. , Zecchin, B. , Terregino, C. , & Scicluna, M. T. (2023). Seroconversion of a Swine Herd in a Free‐Range Rural Multi‐Species Farm against HPAI H5N1 2.3.4.4b Clade Virus. Microorganisms, 11(5), 1162. 10.3390/microorganisms11051162 PMC10224318 37317136

[efs210369-bib-0097] SAG (Servicio Agrícola y Ganadero) , online. SAG confirms first case of highly pathogenic avian influenza in a wild bird in the Coquimbo Region. Available online: https://www.sag.gob.cl/noticias/sag-confirma-primer-caso-de-influenza-aviar-altamente-patogena-en-ave-silvestre-en-la-region-de-coquimbo [Accessed: 10 September 2026]

[efs210369-bib-0098] Saigon, online . Outbreak of H5N1 avian influenza in Dong Thap. Available online: https://www.sggp.org.vn/xuat-hien-o-dich-cum-gia-cam-h5n1-tai-dong-thap-post858716.html [Accessed: 10 September 2026]

[efs210369-bib-0099] Schülein, A. , Ritzmann, M. , Christian, J. , Schneider, K. , & Neubauer‐Juric, A. (2021). Exposure of wild boar to Influenza A viruses in Bavaria: Analysis of seroprevalences and antibody subtype specificity before and after the panzootic of highly pathogenic avian influenza viruses A (H5N8). Zoonoses and Public Health, 68(5), 503–515. 10.1111/zph.12841 33987931

[efs210369-bib-0100] Shin, D. , Siebert, U. , Lakemeyer, J. , Grilo, M. , Pawliczka, I. , Wu, N. , Valentin‐Weigand, P. , Haas, L. , & Herrler, G. (2019). Highly Pathogenic Avian Influenza A(H5N8) Virus in Gray Seals, Baltic Sea. Emerging Infectious Diseases, 25(12), 2295–2298. 10.3201/eid2512.181472 PMC6874272 31742519

[efs210369-bib-0101] Shu, Y. , & McCauley, J. (2017). GISAID: Global initiative on sharing all influenza data – from vision to reality. Euro Surveillance, 22(13). 10.2807/1560-7917.es.2017.22.13.30494 PMC5388101 28382917

[efs210369-bib-0102] Smith, G. J. D. , & Donis, R. O. (2015). Nomenclature updates resulting from the evolution of avian influenza A(H5) virus clades 2.1.3.2a, 2.2.1, and 2.3.4 during 2013–2014. Influenza and Other Respiratory Viruses, 9(5), 271–276. 10.1111/irv.12324 PMC4548997 25966311

[efs210369-bib-0103] Söderquist, P. , Byrne, A. , Lewis, N. , van Toor, M. L. , Waldenström, J. , & SENTINEL Wild Birds Consortium . (2026). SENTINEL Wild Birds yearly report 2025‐2026. Zenodo 10.5281/zenodo.22864464

[efs210369-bib-0104] Suttie, A. , Deng, Y. , Greenhill, A. R. , Dussart, P. , Horwood, P. F. , & Karlsson, E. A. (2019). Inventory of molecular markers affecting biological characteristics of avian influenza A viruses. Virus Genes, 55(6), 739–768. 10.1007/s11262-019-01700-z PMC6831541 31428925

[efs210369-bib-0105] Szaluś‐Jordanow, O. , Golke, A. , Dzieciątkowski, T. , Czopowicz, M. , Kardas, M. , Mickiewicz, M. , Moroz‐Fik, A. , Łobaczewski, A. , Markowska‐Daniel, I. , & Frymus, T. (2024). Upper Respiratory Tract Disease in a Dog Infected by a Highly Pathogenic Avian A/H5N1 Virus. Microorganisms, 12, 689. 10.3390/microorganisms12040689 PMC11051868 38674633

[efs210369-bib-0106] Taft, A. S. , Ozawa, M. , Fitch, A. , Depasse, J. V. , Halfmann, P. J. , Hill‐Batorski, L. , Hatta, M. , Friedrich, T. C. , Lopes, T. J. S. , Maher, E. A. , Ghedin, E. , Macken, C. A. , Neumann, G. , & Kawaoka, Y. (2015). Identification of mammalian‐adapting mutations in the polymerase complexof an avian H5N1 influenza virus. Nature Communications, 6, 7491. 10.1038/ncomms8491 PMC4557292 26082035

[efs210369-bib-0107] Takizawa, N. , & Momose, F. (2022). A novel E198K substitution in the PA gene of influenza A virus with reduced susceptibility to baloxavir acid. Archives of Virology, 167(1), 1565–1570. 10.1007/s00705-022-05456-0 PMC9069958 35511288

[efs210369-bib-0108] Tan, K. , Arshad, N. , Butt, S. L. , Lee, S. , Seo, Y. , Rana, A. M. , Kim, B. , Lee, C. , & Lee, D. (2026). Genetic characterization and zoonotic potential of G1‐Lineage H9N2 avian influenza viruses isolated from poultry in Pakistan, 2023. Avian Pathology., 1–12. 10.1080/03079457.2026.2699158 42566313

[efs210369-bib-0109] TF1 Info , online. Bird flu: the number of infected cats in France is worrying scientists. Available online: https://www.tf1info.fr/sante/video-reportage-grippe-aviaire-le-nombre-de-chats-infectes-en-france-preoccupe-les-scientifiques-2342659.html [Accessed: 28 November 2025]

[efs210369-bib-0110] The Guardian , online‐a. More than 1,000 birds found dead in three mass mortality events amid ‘significant’ H5 escalation in Australia. Available online: https://www.theguardian.com/world/2026/aug/20/more-than-1000-birds-found-dead-in-three-mass-mortality-events-amid-significant-h5-escalation-in-australia [Accessed: 10 September 2026a]

[efs210369-bib-0111] The Guardian , online‐b. Fur seal's death marks first Australian mainland mammal fatality from spreading H5 bird flu strain. Available online: https://www.theguardian.com/world/2026/aug/23/fur-seal-death-first-australian-mainland-mammal-h5-bird-flu [Accessed: 10 September 2026b]

[efs210369-bib-0112] The Kathmandu Post , online. Bird flu hits 162 poultry farms; Rs507 million paid in compensation. Available online: https://kathmandupost.com/money/2026/07/23/bird-flu-hits-162-poultry-farms-rs507-million-paid-in-compensation [Accessed: 10 September 2026]

[efs210369-bib-0113] The Mainichi , online. Bird flu suspected in mass deaths of sea animals in eastern Hokkaido. Available online: https://mainichi.jp/english/articles/20250507/p2a/00m/0li/020000c [Accessed: 10 September 2026]

[efs210369-bib-0114] The Maitland Mercury , online. Bird flu testing, as mass mortality incident detected. Available online: https://www.maitlandmercury.com.au/story/9321824/bird-flu-testing-as-mass-mortality-incident-detected/ [Accessed: 10 September 2026]

[efs210369-bib-0115] The Times of India , online‐a. Red alert as bird flu kills 3 tigers, leopard at Nagpur rescue hub. Available online: https://timesofindia.indiatimes.com/india/red-alert-as-bird-flu-kills-3-tigers-leopard-at-nagpur-rescue-hub/articleshow/116950605.cms [Accessed: 28 November 2025a]

[efs210369-bib-0116] The Times of India , online‐b. Ailing Asiatic lion Pataudi dies amid bird flu scare. Available online: https://timesofindia.indiatimes.com/city/lucknow/ailing-asiatic-lion-pataudi-dies-amid-bird-flu-scare/articleshow/121194380.cms [Accessed: 28 November 2025b]

[efs210369-bib-0117] The Times of India , online‐c. Chhindwara bans meat sales after cat samples test positive for H5N1. Available online: https://timesofindia.indiatimes.com/city/bhopal/chhindwara-bans-meat-sales-after-cat-samples-test-positive-for-h5n1/articleshow/118155020.cms [Accessed: 28 November 2025c]

[efs210369-bib-0118] Tiempo , online. Two cases of avian flu detected at a zoo in Chiapas. Available online: https://www.tiempo.com.mx/nacional/chiapas-detectar-dos-casos-gripe-aviar-h7n3-zoologico-el-zapotal/ [Accessed: 10 September 2026]

[efs210369-bib-0119] UOL Notícias ﻿, online. São Paulo Confirms Second Case of Avian Flu This Year in a Bird Found in Ibirapuera Park. Available online: https://www.facebook.com/photo?fbid=1485063793649497&set=pcb.1485064010316142 [Accessed: 10 September 2026]

[efs210369-bib-0120] USDA (United States Department of Agriculture) , online‐a. Confirmations of Highly Pathogenic Avian Influenza in Commercial and Backyard Flocks. Available online: https://www.aphis.usda.gov/livestock-poultry-disease/avian/avian-influenza/hpai-detections/commercial-backyard-flocks [Accessed: 10 September 2026a]

[efs210369-bib-0121] USDA (United States Department of Agriculture) , online‐b. Detections of Highly Pathogenic Avian Influenza in Wild Birds. Available online: https://www.aphis.usda.gov/livestock-poultry-disease/avian/avian-influenza/hpai-detections/wild-birds [Accessed: 10 September 2026b]

[efs210369-bib-0122] USDA (United States Department of Agriculture) , online‐c. HPAI Confirmed Cases in Livestock. Available online: https://www.aphis.usda.gov/livestock-poultry-disease/avian/avian-influenza/hpai-detections/hpai-confirmed-cases-livestock [Accessed: 10 September 2026c]

[efs210369-bib-0123] USDA (United States Department of Agriculture) , online‐d. Detections of Highly Pathogenic Avian Influenza in Mammals. Available online: https://www.aphis.usda.gov/livestock-poultry-disease/avian/avian-influenza/hpai-detections/mammals?page=1 [Accessed: 10 September 2026d]

[efs210369-bib-0124] Vilas Radio , online. Alert in Iquique: SAG confirms first outbreak of highly pathogenic avian influenza in wild bird. Available online: https://vilasradio.cl/alerta-en-iquique-sag-ratifica-primer-foco-de-influenza-aviar-altamente-patogena-en-ave-silvestre/ [Accessed: 10 September 2026]

[efs210369-bib-0125] Vu, M. N. , Quirk, G. E. , Smathers, A. E. , Busfield‐Thomason, K. , Dorazio, A. L. , Domke, K. R. , Humber, G. M. , Sembrat, J. , McElroy, A. K. , Le Sage, V. , & Lakdawala, S. S. (2026). A lethal human H5N5 influenza virus isolate exhibits low pandemic risk traits. bioRxiv, 2026(7). Advance online publication. 10.64898/2026.07.20.739507

[efs210369-bib-0126] WHO (World Health Organization) , 2023. Genetic and antigenic characteristics of zoonotic influenza A viruses and development of candidate vaccine viruses for pandemic preparedness Geneva, 12 pp. Available online: https://cdn.who.int/media/docs/default-source/influenza/who-influenza-recommendations/vcm-northern-hemisphere-recommendation-2023-2024/20230224_zoonotic_recommendations.pdf [Accessed: 10 September 2026]

[efs210369-bib-0127] WHO (World Health Organization) , online‐a. Avian Influenza Weekly Update Number 957. Available online: https://iris.who.int/server/api/core/bitstreams/a6160669-41f8-4f36-980f-d4ba470c075d/content [Accessed: 10 September 2026a]

[efs210369-bib-0128] WHO (World Health Organization) , online‐b. Cumulative number of confirmed human cases for avian influenza A(H5N1) reported to WHO, 2003‐2026. Available online: https://cdn.who.int/media/docs/default-source/influenza/h5n1-human-case-cumulative-table/cumulative-number-of-confirmed-human-cases-for-avian-influenza-a(h5n1)-reported-to-who--2003-20267f2be8b2-0de2-43f6-9d81-7d7c8c2b7670.pdf [Accessed: 10 September 2026b]

[efs210369-bib-0129] WHO (World Health Organization) , online‐c. Influenza at the human‐animal interface. Summary and risk assessment, from 13 June to 7 July 2026. Available online: https://cdn.who.int/media/docs/default-source/influenza/human-animal-interface-risk-assessments/influenza-at-the-human-animal-interface-summary-and-assessment--from-13-june-to-7-july-2026.pdf [Accessed: 10 September 2026c]

[efs210369-bib-0130] WHO (World Health Organization) , online‐d. Influenza at the human‐animal interface. Summary and risk assessment, from 8 July to 7 August 2026. Available online: https://cdn.who.int/media/docs/default-source/influenza/human-animal-interface-risk-assessments/influenza-at-the-human-animal-interface-summary-and-assessment--from-8-july-to-7-august-2026.pdf [Accessed: 10 September 2026d]

[efs210369-bib-0131] WHO (World Health Organization) , online‐e. Avian Influenza Weekly Update Number 1053. Available online: https://cdn.who.int/media/docs/default-source/wpro---documents/emergency/surveillance/avian-influenza/ai_20260718.pdf [Accessed: 10 September 2026e]

[efs210369-bib-0132] WHO (World Health Organization) , online‐f. Influenza at the human‐animal interface. Summary and risk assessment, from 9 May to 12 June 2026. Available online: https://cdn.who.int/media/docs/default-source/_sage-2026/influenza-at-the-human-animal-interface-summary-and-assessment--from-9-may-to-12-june-2026.pdf [Accessed: 10 September 2026f]

[efs210369-bib-0133] WHO (World Health Organization) , online‐g. Avian Influenza Weekly Update Number 1055. Available online: https://cdn.who.int/media/docs/default-source/wpro---documents/emergency/surveillance/avian-influenza/ai_20260731.pdf [Accessed: 10 September 2026g]

[efs210369-bib-0134] WHO (World Health Organization) , online‐h. Avian Influenza Weekly Update Number 1049. Available online: https://cdn.who.int/media/docs/default-source/wpro---documents/emergency/surveillance/avian-influenza/ai_20260619.pdf [Accessed: 10 September 2026h]

[efs210369-bib-0135] WHO (World Health Organization) , online‐i. Avian Influenza Weekly Update Number 1057. Available online: https://cdn.who.int/media/docs/default-source/wpro---documents/emergency/surveillance/avian-influenza/ai_20260814.pdf [Accessed: 10 September 2026i]

[efs210369-bib-0136] WHO (World Health Organization) , online‐j. Avian Influenza Weekly Update Number 1058. Available online: https://cdn.who.int/media/docs/default-source/wpro---documents/emergency/surveillance/avian-influenza/ai_20260821.pdf [Accessed: 10 September 2026j]

[efs210369-bib-0137] WHO (World Health Organization) , online‐k. Avian Influenza Weekly Update Number 1059. Available online: https://cdn.who.int/media/docs/default-source/wpro---documents/emergency/surveillance/avian-influenza/ai_20260828.pdf [Accessed: 10 September 2026k]

[efs210369-bib-0138] WHO (World Health Organization) , online‐l. Avian Influenza A(H5N1) ‐ México. Available online: https://www.who.int/emergencies/disease-outbreak-news/item/2025-DON564 [Accessed: 10 September 2026l]

[efs210369-bib-0139] Xinhua News , online. New Zealand confirms 3rd H5 bird flu case. Available online: https://english.news.cn/20260821/986e3af6df2b4c9a97b20d70aeec05cd/c.html [Accessed: 10 September 2026]

[efs210369-bib-0140] Yamada, S. , Hatta, M. , Staker, B. L. , Watanabe, S. , Imai, M. , Shinya, K. , Sakai‐Tagawa, Y. , Ito, M. , Ozawa, M. , Watanabe, T. , Sakabe, S. , Li, C. , Kim, J. H. , Myler, P. J. , Phan, I. , Raymond, A. , Smith, E. , Stacy, R. , Nidom, C. A. , … Kawaoka, Y. (2010). Biological and Structural Characterization of a Host‐Adapting Amino Acid in Influenza Virus. PloS Pathogens, 6(8), e1001034. 10.1371/journal.ppat.1001034 PMC2916879 20700447

[efs210369-bib-0141] Yao, X. , Lian, C. , Lv, Z. , Zhang, X. , & Shao, J. (2023). Emergence of a novel reassortant H5N6 subtype highly pathogenic avian influenza virus in farmed dogs in China. Journal of Infection, 87(4), e70–e72. 10.1016/j.jinf.2023.07.013 37507094

[efs210369-bib-0142] Youk, S. , Torchetti, M. K. , Lantz, K. , Lenoch, J. B. , Killian, M. L. , Leyson, C. , Bevins, S. N. , Dilione, K. , Ip, H. S. , Stallknecht, D. E. , Poulson, R. L. , Suarez, D. L. , Swayne, D. E. , & Pantin‐Jackwood, M. J. (2023). H5N1 highly pathogenic avian influenza clade 2.3.4.4b in wild and domestic birds: Introductions into the United States and reassortments, December 2021–April 2022. Virology., 587, 109860. 10.1016/j.virol.2023.109860 37572517

[efs210369-bib-0143] Zhao, J. , Wan, W. , Yu, K. , Lemey, P. , Pettersson, J. H. , Bi, Y. , Lu, M. , Li, X. , Chen, Z. , Zheng, M. , Yan, G. , Dai, J. , Li, Y. , Haerheng, A. , He, N. , Tu, C. , Suchard, M. A. , Holmes, E. C. , He, W. , & Su, S. (2024). Farmed fur animals harbour viruses with zoonotic spillover potential. Nature, 634, 228–233. 10.1038/s41586-024-07901-3 PMC11741233 39232170

